# DNA methylation and gene expression dynamics during spermatogonial stem cell differentiation in the early postnatal mouse testis

**DOI:** 10.1186/s12864-015-1833-5

**Published:** 2015-08-20

**Authors:** Naoki Kubo, Hidehiro Toh, Kenjiro Shirane, Takayuki Shirakawa, Hisato Kobayashi, Tetsuya Sato, Hidetoshi Sone, Yasuyuki Sato, Shin-ichi Tomizawa, Yoshinori Tsurusaki, Hiroki Shibata, Hirotomo Saitsu, Yutaka Suzuki, Naomichi Matsumoto, Mikita Suyama, Tomohiro Kono, Kazuyuki Ohbo, Hiroyuki Sasaki

**Affiliations:** Division of Epigenomics and Development, Medical Institute of Bioregulation, Kyushu University, Fukuoka, 812-8582 Japan; Research Institute for Disease of the Chest, Graduate School of Medical Sciences, Kyushu University, Fukuoka, 812-8582 Japan; Department of Histology and Cell Biology, Yokohama City University School of Medicine, Yokohama, 236-0004 Japan; NODAI Genome Research Center, Tokyo University of Agriculture, Tokyo, 156-8502 Japan; Division of Bioinformatics, Medical Institute of Bioregulation, Kyushu University, Fukuoka, 812-8582 Japan; Department of Human Genetics, Graduate School of Medicine, Yokohama City University, Yokohama, 236-0004 Japan; Division of Genomics, Medical Institute of Bioregulation, Kyushu University, Fukuoka, 812-8582 Japan; Department of Medical Genome Sciences, Graduate School of Frontier Sciences, The University of Tokyo, Kashiwanoha 5-1-5, Kashiwa, Chiba 277-8568 Japan; Department of BioScience, Tokyo University of Agriculture, Tokyo, 156-8502 Japan

**Keywords:** DNA methylation, Non-CG methylation, 5-hydroxymethylcytosine, Spermatogenesis, Spermatogonial stem cell, Prospermatogonia, Spermatogonia

## Abstract

**Background:**

In the male germline, neonatal prospermatogonia give rise to spermatogonia, which include stem cell population (undifferentiated spermatogonia) that supports continuous spermatogenesis in adults. Although the levels of DNA methyltransferases change dynamically in the neonatal and early postnatal male germ cells, detailed genome-wide DNA methylation profiles of these cells during the stem cell formation and differentiation have not been reported.

**Results:**

To understand the regulation of spermatogonial stem cell formation and differentiation, we examined the DNA methylation and gene expression dynamics of male mouse germ cells at the critical stages: neonatal prospermatogonia, and early postntal (day 7) undifferentiated and differentiating spermatogonia. We found large partially methylated domains similar to those found in cancer cells and placenta in all these germ cells, and high levels of non-CG methylation and 5-hydroxymethylcytosines in neonatal prospermatogonia. Although the global CG methylation levels were stable in early postnatal male germ cells, and despite the reported scarcity of differential methylation in the adult spermatogonial stem cells, we identified many regions showing stage-specific differential methylation in and around genes important for stem cell function and spermatogenesis. These regions contained binding sites for specific transcription factors including the SOX family members.

**Conclusions:**

Our findings show a distinctive and dynamic regulation of DNA methylation during spermatogonial stem cell formation and differentiation in the neonatal and early postnatal testes. Furthermore, we revealed a unique accumulation and distribution of non-CG methylation and 5hmC marks in neonatal prospermatogonia. These findings contrast with the reported scarcity of differential methylation in adult spermatogonial stem cell differentiation and represent a unique phase of male germ cell development.

**Electronic supplementary material:**

The online version of this article (doi:10.1186/s12864-015-1833-5) contains supplementary material, which is available to authorized users.

## Background

In mammalian spermatogenesis, huge numbers of spermatozoa are produced throughout adult life. This constant supply is supported by the spermatogonial stem cell (SSC) system [1–3]. In male mouse embryos, primordial germ cells become arrested at the G1/G0 phase of the cell cycle around embryonic day 13 (E13), giving rise to prospermatogonia (PSGs, also known as gonocytes). The PSGs resume mitosis after birth, resulting in the formation of spermatogonia (SGs) [4]. Although the first round of spermatogenesis skips the stem cell stage [5], the mouse testis at postnatal day 7 (P7) already contains both undifferentiated and differentiating SGs [5, 6], the former of which are thought to include the initial SSC population [7]. Some cytological markers such as Kit (also known as c-Kit) are available to distinguish between undifferentiated and differentiating SGs [7–12]. Only Kit-negative (Kit^−^) SGs contain the SSC population (the undifferentiated SGs), and Kit-positive (Kit^+^) SGs are the differentiating SGs, where Kit is important for migration, proliferation, and differentiation [13–15]. However, the mechanisms involved in the genetic and epigenetic regulation of SSC formation and differentiation in early postnatal testis are largely unknown.

It is well established that DNA methylation is important for germ cell development [16]. In fetal PSGs, the de novo DNA methyltransferase DNMT3A and its cofactor DNMT3L are expressed highly, and paternally methylated imprinting control regions (ICRs) and many retrotransposons are methylated de novo [17–19]. Thus, neonatal PSGs have a high level of genome-wide methylation. Targeted disruption of the *Dnmt3a* or *Dnmt3l* genes in the male germline results in developmental arrest at the spermatocyte stage and subsequent loss of germ cells, indicating an essential role of methylation in spermatogenesis [20, 21]. However, the detailed methylation profile of neonatal PSGs has not been reported. Furthermore, it is totally unknown how DNA methylation and gene expression profiles change during the transitions from PSGs to undifferentiated SGs and from undifferentiated to differentiating SGs in early postnatal testis.

Despite the lack of molecular studies, interesting cytological observations have been made. First, an immunofluorescence study using an anti-5-methylcytosine (5mC) antibody showed that chromosome arms lose staining in a replication-dependent way upon the transition from neonatal PSGs to SGs [22]. Interestingly, the centromeric regions were barely stained in these cells. Second, increased production of DNMT3A and DNMT3B was observed during the transition from undifferentiated to differentiating SGs in early postnatal and adult testes [23]. It was also shown that undifferentiated SGs were less intensely stained for 5mC than were differentiating SGs. Thus, there might be an epigenetic switch important for the transition from undifferentiated to differentiating SGs [23]. However, a recent whole-genome bisulfite sequencing (WGBS) study showed that methylation differences are rather rare between undifferentiated (Thy1^+^) and differentiating (Kit^+^) SGs in adult testis and that only a few promoter regions show differences [24].

Here, we have determined the DNA methylation and gene expression profiles of highly purified neonatal PSGs and early postnatal SGs by WGBS and RNA sequencing (RNA-seq). We used expression of an *Oct4*-driven transgene as a marker for germ cells, and Kit as a marker for differentiating SGs. Our WGBS revealed unique distributions of 5mC and 5-hydroxymethylcytosine (5hmC) and transient accumulation of non-CG methylation. Furthermore, we identified genomic regions showing stage-specific changes in CG methylation that were closely associated with genes having roles in stem cell function, cell proliferation, and spermatogenesis. These regions are rich in binding sites for specific transcription factors and likely represent important regulatory elements. Our results provide insights into the epigenetic regulation of SSC formation and differentiation.

## Results

### WGBS and RNA-seq of neonatal PSGs and early postnatal SGs

**Fig. 1:**
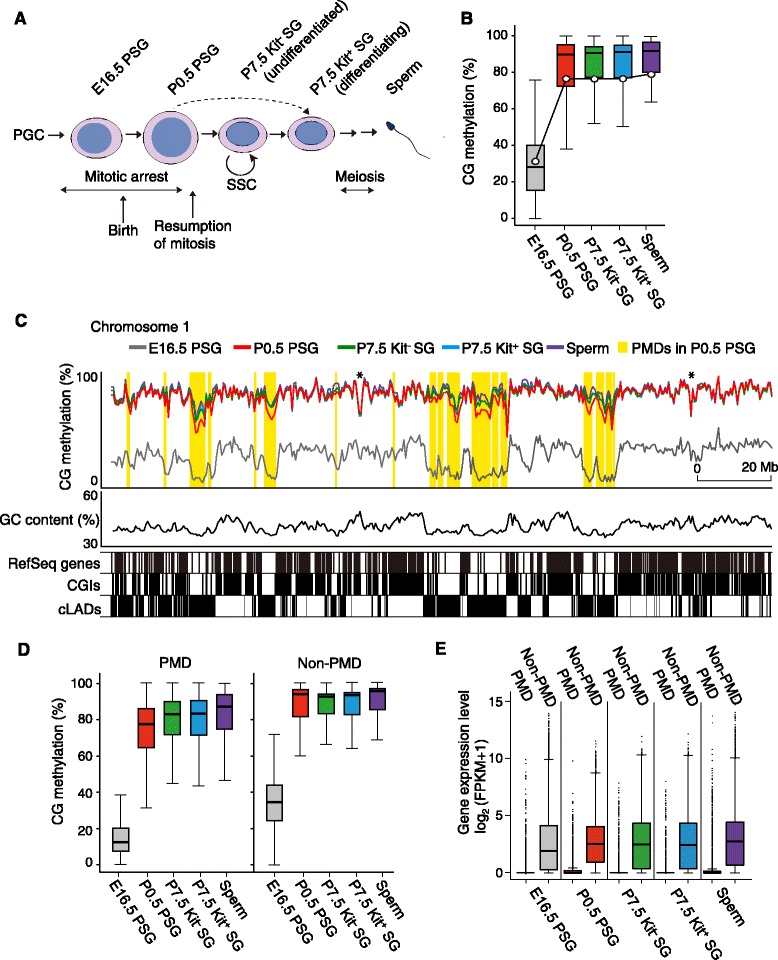
Overall CG methylation levels and distribution. **a** Schematic representation of male germ cell development and the cell types analyzed in this study. The interrupted arrow shows that the first round of spermatogenesis skips the stem cell stage. PGC, primordial germ cell. **b** Developmental changes in the level of CG methylation in the entire genome (*line graph*) and in 10 kb windows (*box plots*). Central bar, median; lower and upper box limits, 25th and 75th percentiles, respectively; whiskers, 1.5 times the interquartile range from the 25th to the 75th percentile. The E16.5 PSG data are from Kobayashi et al. [18]. **c** Distribution of CG methylation across mouse chromosome 1. The methylation levels determined in 500 kb windows are shown for the respective cell types (*colored lines*). Large PMDs (≥500 kb) identified in P0.5 PSGs by MethylSeekR are highlighted in yellow. The GC contents in 500 kb windows are also shown. cLADs are lamina-associated domains common to embryonic stem cells, neural precursor cells, astrocytes, and embryonic fibroblasts [39]. The CG methylation valleys marked with asterisks are not PMDs but composites of unmethylated CGIs and hypermethylated segments. **d** Developmental changes in the CG methylation level in 10 kb windows in PMDs and non-PMDs. Those windows overlapping with any PMDs in P0.5 PSGs were considered as PMDs and the rest were considered as non-PMDs. **e** Transcript levels of genes located in PMDs and non-PMDs. The transcript levels are shown as log_2_ (FPKM + 1). The PMD genes are RefSeq coding genes whose promoter regions (from 2000 bp upstream to 500 bp downstream of the transcription start site) overlap with any PMDs. The remaining genes are non-PMD

PSGs arrested at the G1/G0 phase resume mitosis in the neonatal testis and give rise to undifferentiated and differentiating SGs by the end of the first week [4, 25]. The differentiation of SGs coincides with the expression of Kit, and only Kit^−^ SGs contain SSCs [13–15]. We isolated germ cells from transgenic mice carrying an *Oct4*-driven enhanced green fluorescent protein (EGFP) gene based on the expression of the transgene (germ cell marker) and expression of endogenous Kit (differentiation marker). The isolated cells were P0.5 PSGs (*Oct4*-EGFP^+^), and undifferentiated (*Oct4*-EGFP^+^, Kit^−^) and differentiating P7.5 SGs (*Oct4*-EGFP^+^, Kit^+^) (Fig. 1a).

To construct WGBS libraries, we used the post-bisulfite adaptor tagging (PBAT) method, which enables amplification-free library construction from a minute amount of DNA [26]. We obtained > 691 million uniquely mapped reads for each sample, with an average depth of > 15.5 × per strand, or > 31.0 × per DNA molecule, and a CG coverage of 94.3–94.4 % (Additional file 1: Table S1). For each cell type, independent preparations (biological replicates) were studied to confirm the reproducibility (R > 0.953). All samples were spiked with unmethylated lambda DNA to calculate the bisulfite conversion efficiency, which always exceeded 99.5 %. This conversion rate is sufficient for the analysis of non-CG (or CH) methylation [27]. For gene expression profiling, poly(A)^+^ RNAs from replicate samples were analyzed by RNA-seq (R > 0.981). We normally obtained > 83 million reads for each sample (Additional file 1: Table S1). For comparison, we processed existing bisulfite sequencing data from E16.5 PSGs [15] and RNA-seq data from adult spermatozoa [28], but also performed additional sequencing with our own samples (Additional file 1: Table S1).

### Distinctive gene expression signatures

We first examined the expression of known molecular markers (Additional file 2: Figure S1). We confirmed that *Oct4* and *Kit* showed the expected expression patterns. Genes highly expressed in E16.5 PSGs such as *Dnmt3a* and *Dnmt3l* showed consistent expression in P0.5 PSGs, but *Nanos2* was downregulated. SSC markers such as *Plzf*, *Gfra1*, *Ngn3, Id4,* and *Nanos2* [29] were expressed in P7.5 Kit^−^ SGs and downregulated in Kit^+^ SGs. Genes involved in the signal transduction pathways for SSC self-renewal such as *Foxo1*, *Etv5*, *Ret*, *Bcl6b, T* (also known as *Brachyury*), and *Cxcr4* [29] were also downregulated in Kit^+^ SGs. Expression of *Thy1* was not high in either cell type. In contrast, Kit^+^ SGs expressed *Stra8*, consistent with its role in committing SGs to meiosis. In contrast with our previous cytology finding [23], only *Dnmt3b* (but not *Dnmt3a*) was upregulated in Kit^+^ SGs. All preparations were virtually negative for the Sertoli cell markers *Gata4* and *Gata6* [30] and the Leydig cell markers *Cyp11a1* and *Hsd3b1* [31] (Additional file 2: Figure S1). The DNA methylation levels of the germline ICRs [32] also supported negligible somatic contamination. More specifically, in contrast to the 50 % methylation level expected for somatic cells, the paternally methylated ICRs showed high methylation (>80 %), whereas the maternally methylated ICRs showed low methylation (<10 %) (Additional file 2: Figure S2A). Overall, our results are consistent with the known distinctive signatures of these prepared cell types.

### Large partially methylated domains

We first compared the overall CG methylation profiles (Fig. 1b). The methylation level increased from 30.1 % in E16.5 PSGs to 76.1 % in P0.5 PSGs, but it did not change much in P7.5 Kit^−^ and Kit^+^ SGs (76.6 % and 76.4 %, respectively) (Fig. 1b). The final methylation level in adult spermatozoa was 79.1 %. When the methylation level was calculated in nonoverlapping 50 kb windows, the median value increased consistently from 28.0 % in E16.5 PSGs to 93.9 % in adult spermatozoa, with E16.5 PSGs showing the widest distribution (Fig. 1b).

Interestingly, we found large genomic domains (up to 12.0 Mb) with relative hypomethylation in all cell types (Fig. 1c; Additional file 2: Figure S3). These domains resembled the partially methylated domains (PMDs) reported in cultured fibroblast cells [33, 34], cancer cells [35, 36], and placenta [37]. The PMDs identified in P0.5 PSGs using MethylSeekR [38] (only those ≥ 500 kb are shown in Fig. 1c; Additional file 2: Figure S3) were located in genomic regions of low GC content, low CG island (CGI) density, and low gene density, as reported previously for the other cell types. These regions overlapped with constitutive nuclear lamina-associated domains (cLADs) [35, 39], which show late DNA replication during the S phase (Fig. 1c). The existence of PMDs is consistent with the previous observation that whole testis DNA tends to have more hypomethylated CG sites in low GC content regions than in somatic tissues [40]. In addition, some PMDs that we found in X chromosome were identical to the previously reported large hypomethylated domains or LoDs [41] (Additional file 2: Figure S3).

The PMDs were not present when we examined the WGBS data from E10.5 primordial germ cells, which are undergoing demethylation [18]. The PMDs showed a slow but consistent increase in CG methylation thereafter. In contrast, non-PMD regions obtained high levels of methylation more quickly by P0.5 in PSGs and then changed little (Fig. 1d). Interestingly, previous studies showed that genes in the PMDs are silenced in cancer cells, despite their hypomethylated state [35, 36]. Our RNA-seq results confirmed a similar gene silencing effect at all stages (Fig. 1e). Thus, the PMDs identified in germ cells share not only physical features but also functional consequences with the PMDs reported in other cell types.

### High levels of CH methylation in neonatal PSGs

**Fig. 2:**
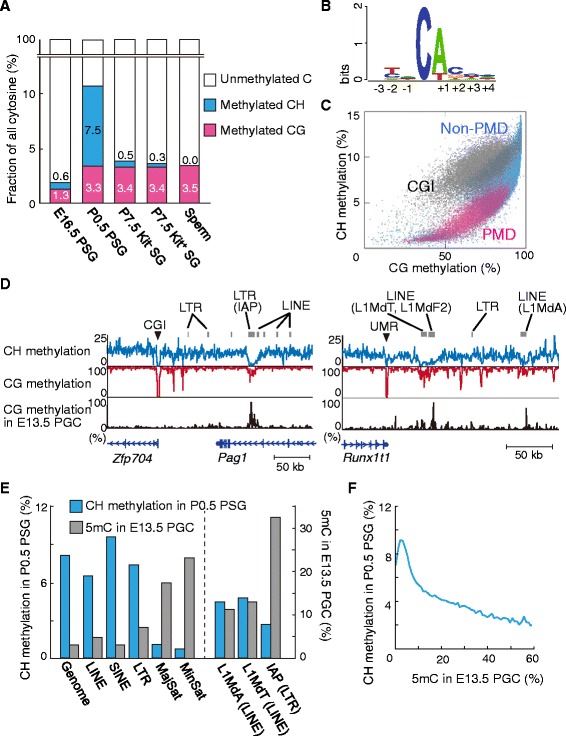
High levels of CH methylation in neonatal PSGs. **a** Proportions of methylated cytosines at CG and CH sites, and unmethylated cytosines at all sites. The nonconversion rate was subtracted from the level of methylated cytosine at CG and CH sites, assuming that it occurred randomly at any unmethylated cytosine. **b** Sequence context of methylated CH in P0.5 PSGs. **c** A scatter plot showing correlations between the levels of CH and CG methylation in P0.5 PSGs. Each dot represents both levels in a 50 kb window. The 50 kb regions were classified into those containing any CGI (almost all of which are non-PMD regions; *gray*) and those without CGI, and the latter were further classified into PMD (*red*) or non-PMD regions (*blue*). **d** Discrepancy between the CG and CH methylation levels observed at some retrotransposons. The CH (*top*) and CG methylation (*middle*) levels in P0.5 PSGs are compared with the CG methylation levels in E13.5 PGCs (*bottom*) across two genomic regions. An LTR (IAP) (*left panel*) and LINE copies (*right panel*) have high levels of CG methylation but very low levels of CH methylation in PSGs. These retrotransposon copies are more CG methylated than the adjacent regions in PGCs. The methylation levels were determined in 500 bp windows. Regions marked with arrowheads are CGI or unmethylated region (UMR). **e** The CH methylation levels in P0.5 PSGs (*blue bar*) and the CG methylation levels in E13.5 PGCs (*gray bar*) in the whole genome and repeat sequences. The levels of the major and minor satellite repeats were calculated from reads aligned to the consensus sequences, while those of the other sequences were calculated from reads uniquely aligned to the reference mouse genome (mm10). **f** Negative correlation between the CH methylation levels in P0.5 PSGs and the CG methylation levels in E13.5 PGCs. The methylation levels were calculated in 5 kb windows

We previously reported the presence of CH (where H = A, C, or T) methylation within and around the short interspersed nuclear element (SINE) B1 in neonatal PSGs [42]. The present study revealed surprisingly high levels of genome-wide CH methylation in P0.5 PSGs. Approximately 10.8 % of all cytosines were methylated in this cell type, compared with 3–4 % in somatic tissues, and more than two-thirds of 5mCs (7.5 % of all cytosines) were at CH sites (Fig. 2a). Conversely, only one-third of 5mCs (3.3 % of all cytosines) were at CG sites in P0.5 PSGs. Among the CH sequences, CA was the most frequently methylated (methylation level, 16.9 %) (Fig. 2b). Overall, genomic regions showing high levels of CG methylation (non-PMD regions) had high levels of CH methylation. Among the non-PMD regions, CGI-containing regions showed a relatively strong correlation between the two levels, but the rest of the non-PMD regions had high CH methylation only when CG methylation was very high (Fig. 2c).

The typical CGIs were devoid of both CG and CH methylation (Fig. 2d). Interestingly, some long interspersed nuclear element (LINE) and long terminal repeat (LTR) retrotransposon copies had low levels of CH methylation, but relatively high levels of CG methylation, with the adjacent regions having high levels of both CG and CH methylation (Fig. 2d). The LINE copies with low CH methylation levels belonged to the evolutionarily younger subfamilies of the L1 family, such as L1MdA and L1MdT (Fig. 2e). Likewise, many of the LTR copies devoid of CH methylation were members of the intracisternal A particle (IAP) family (Fig. 2e), which is again a relatively young class of endogenous retrovirus. It is known that, although the majority of retrotransposons become hypomethylated in primordial germ cells, certain copies stay CG methylated [19], indicating a resistance to reprogramming. Interestingly, the abovementioned LINE and LTR copies, which were devoid of CH methylation, maintained some level of CG methylation in E13.5 primordial germ cells [18] (Fig. 2d–f). Thus, the de novo DNMTs responsible for CH methylation in fact introduce fewer CH methylation marks in regions maintaining residual CG methylation.

### CH methylation explains cytological observations

A previous immunocytological study showed that the chromosome arms of neonatal PSGs are strongly stained for 5mC [22] (Additional file 2: Figure S4). Very interestingly, the same study showed that the signals are then lost in a replication-dependent way in early postnatal SGs. Our findings now suggest that the observed signal loss might be caused by the loss of CH methylation, but not of CG methylation, because the CH methylation level is more than 2-fold higher than the CG methylation level in neonatal PSGs (Fig. 2a) and there is no maintenance mechanism for CH methylation in mammals. Indeed, CH methylation was lost rapidly after the resumption of mitosis (Fig. 2a). Despite the genome-wide loss of CH methylation, the CG methylation levels did not change much in the postnatal SGs (Figs. 1b and 2a). More specifically, high levels of CG methylation (>80 %) were maintained at the *H19*, *Rasgrf1*, and *Dlk1-Gtl2* ICRs in P7.5 Kit^−^ and Kit^+^ SGs (Additional file 2: Figure S2B). Retrotransposons also maintained high levels of CG methylation, although satellite repeats showed lower methylation levels (Additional file 2: Figure S2C). Consistent with the CG methylation maintenance, genes coding for proteins involved in methylation maintenance, such as *Dnmt1* and *Uhrf1*, were expressed in these cells (Additional file 2: Figure S2D).

### High levels of 5hmC in satellite repeats of neonatal PSGs

**Fig. 3:**
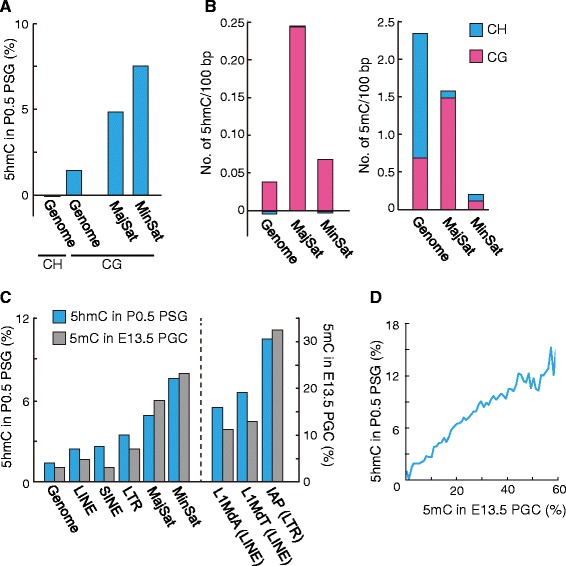
Distribution of 5hmC in P0.5 PSGs. **a** 5hmC levels in the whole genome and satellite repeats. The levels in the major and minor satellite repeats were calculated from sequence reads aligned to the consensus sequences. The levels in the whole genome were calculated from reads uniquely aligned to the reference mouse genome (mm10). **b** Densities of 5hmC and 5mC (per 100 bp) in the whole genome and major and minor satellite repeats. The proportions of 5hmCs/5mCs at CG and CH sites are also shown. **c** The 5hmC levels of the whole genome and repeat sequence in P0.5 PSGs (*blue bar*) and the 5mC levels of those in E13.5 PGCs (*gray bar*). The levels of the satellite repeats were calculated from reads aligned to the consensus sequences, while those of the other sequences were calculated from reads uniquely aligned to the reference mouse genome (mm10). **d** Positive correlation between the 5hmC levels in P0.5 PSGs and the 5mC levels in E13.5 PGCs. The levels were calculated in 5 kb windows

5hmC has been recognized as an important intermediate for demethylation [43], and its levels and distribution change during spermatogenesis [44]. The abovementioned cytological study showed that the centromeric and pericentromeric regions are only weakly stained for 5mC in neonatal PSGs and early postnatal SGs [22] (Additional file 2: Figure S4). This was attributed to hydroxylation of 5mCs in recent studies using anti-5hmC antibodies [45, 46] (Additional file 2: Figure S4). To test this at the molecular level, the 5hmC profile of P0.5 PSGs was determined by oxidative bisulfite sequencing [47] combined with the PBAT method. Almost all 5hmCs occurred at CG sites, consistent with a previous study on embryonic stem cells [48], and the genome-wide 5hmC level was 1.4 % (Fig. 3a). As expected, the minor and major satellite repeats, corresponding to the centromeric and pericentromeric regions, respectively, showed higher 5hmC levels (7.5 % and 4.8 %, respectively) than the whole genome (Fig. 3a). When the frequency of the modified base per length was determined, the major satellites showed the highest 5hmC density, whereas the whole genome showed the highest 5mC density (Fig. 3b), consistent with the immunocytological observations. The high level of 5hmC at the satellite repeats might be important for silencing these repeats in PSGs [46] and, after this stage, the 5hmC signals disappear in a replication-dependent way [45, 46] (Additional file 2: Figure S4). Among the retrotransposons, the IAP family and the younger subfamilies of L1 had high levels of 5hmC (Fig. 3c). As mentioned above, these retrotransposon species are resistant to reprogramming and retain some levels of CG methylation in E13.5 primordial germ cells (Fig. 3c and d).

### Stage-specific differentially methylated regions (DMRs)

We next sought to identify regions showing developmental stage-specific changes in CG methylation because cell-type-specific local hypomethylation often marks active regulatory regions [49]. The CG methylation levels were compared in 500 bp windows (≥5 CG sites, 100 bp sliding steps) between P0.5 PSGs and P7.5 Kit^−^ or between P7.5 Kit^−^ and Kit^+^ SGs, and regions spanned by overlapping windows showing > 30 % CG methylation differences in either sample pair were identified. As a result, we found a total of 5387 DMRs (500–2400 bp) (*P* < 0.02, false discovery rate (FDR) < 0.05), which together constituted 0.14 % of the genome. Thus, despite the reported scarcity of methylation differences between undifferentiated (Thy1^+^) and differentiating (Kit^+^) adult SGs [24], we identified many DMRs between the undifferentiated and differentiating early postnatal SGs (see below). On the other hand, our DMRs did not include any of the seven promoters showing > 30 % CG methylation difference between the undifferentiated and differentiating adult SGs [24].

**Fig. 4:**
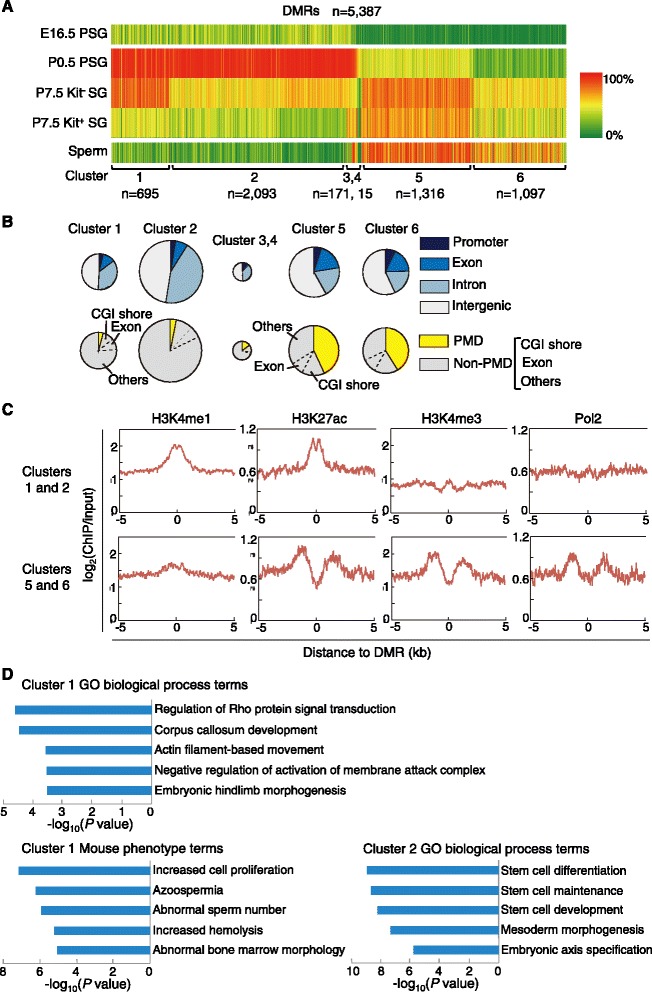
Identification and characterization of stage-specific DMRs. **a** A heat-map representation of the changes in CG methylation at the DMRs identified in the transitions from P0.5 PSGs to P7.5 Kit^−^ SGs and from P7.5 Kit^−^ SGs to Kit^+^ SGs. The color gradation (*from green to red*, *with an intermediate in yellow*) shows the methylation levels (*from low to high*). The six DMR groups identified by cluster analysis using the changing methylation patterns are shown. The methylation levels in E16.5 PSGs and mature spermatozoa are also shown for comparison. **b** Genomic locations of the DMRs of each cluster relative to the gene structure (*top*) or PMD/non-PMD regions (*bottom*). The DMRs in non-PMD regions are further subdivided based on whether they overlap with CGI shores (2 kb from the edge of a CGI) or exons. The circle size represents the number of DMRs. **c** Levels of H3K4me1, H3K4me3, H3K27ac, and RNA polymerase II (Pol2) relative to the DMRs in the adult mouse testis [50]. The data are shown for combined cluster-1 and −2 DMRs and combined cluster-5 and −6 DMRs. **d** GO biological process and mouse phenotype terms enriched for the DMRs of clusters 1 and 2 by GREAT analysis

The DMRs were then subjected to cluster analysis based on the patterns of stage-dependent methylation changes. Six distinct clusters were noted (Fig. 4a; Additional file 3: Table S2). The cluster-1 DMRs (*n* = 695) were highly methylated in P0.5 PSGs and P7.5 Kit^−^ SGs but less methylated in Kit^+^ SGs. Cluster-2 DMRs (*n* = 2093) had high levels of methylation in P0.5 PSGs, but showed decreased methylation in P7.5 Kit^−^ SGs and even lower methylation in Kit^+^ SGs. Clusters 5 and 6 (*n* = 1316 and 1097, respectively) showed low methylation in P0.5 PSGs and higher methylation in P7.5 Kit^+^ SGs, but only cluster 5 showed an increase in Kit^−^ SGs. Clusters 3 and 4 had distinct methylation patterns, with cluster-3 gaining methylation during the transition from Kit^−^ SGs to Kit^+^ SGs, but there were only small numbers of DMRs in these clusters.

Only a small fraction of the DMRs of each cluster was found to overlap with promoters (Fig. 4b), as reported in undifferentiated and differentiating adult SGs [24]. Rather, most DMRs—especially those belonging to clusters 1 and 2—were intergenic or intronic. When published histone modification data from the adult testis [50] were analyzed, the DMRs of clusters 1 and 2 showed enrichment in enhancer marks such as histone H3 lysine-4 monomethylation (H3K4me1) and histone H3 lysine-27 acetylation (H3K27ac) [51, 52] (Fig. 4c), suggesting that they are distal or intronic regulatory regions. However, in clusters 5 and 6, many of the DMRs were mapped to the PMDs (Fig. 4b). Because the genes in PMDs are mostly silent through the stages (Fig. 1e), the observed methylation changes did not seem to be relevant to regulation, but might rather reflect the ongoing de novo methylation of PMDs (Fig. 1c and d). The rest of the DMRs of clusters 5 and 6 coincided with CGI shores or exons in non-PMD regions, of which methylation can be a cause or a consequence of gene activity. However, the DMRs of these clusters lacked histone modification marks for enhancers or promoters (Fig. 4c).

We then sought to correlate the DMRs with specific gene functions based on their genomic locations using Genomic Regions Enrichment of Annotations Tool (GREAT) analysis [53]. This revealed that the DMRs of cluster 1 were significantly correlated with gene ontology (GO) biological processes terms “regulation of Rho protein signal transduction” (*P* = 2.5 × 10^−5^) and “actin filament-based movement” (*P* = 2.4 × 10^−4^) (Fig. 4d; Additional file 4: Table S3), consistent with the active proliferation and movement of differentiating SGs. The DMRs were also associated with mouse phenotype GO terms “cell proliferation” (*P* = 6.6 × 10^−8^), “azoospermia” (*P* = 6.0 × 10^−7^), and “abnormal sperm number” (*P* = 1.2 × 10^−6^) (Fig. 4d; Additional file 4: Table S3). The DMRs of cluster 2 were significantly correlated with the biological process terms “stem cell differentiation” (*P* = 1.1 × 10^−9^), “stem cell maintenance” (*P* = 2.1 × 10^−9^), and “stem cell development” (*P* = 5.7 × 10^−9^) (Fig. 4d; Additional file 4: Table S3), consistent with a role in undifferentiated SGs. In contrast, clusters 5 and 6 did not show significant enrichment (FDR < 0.1) even though the DMR numbers were sufficient for the analysis.

### Correlation between DNA methylation and gene expression profiles

To correlate the DMRs with gene expression, we next identified genes showing > 2.0-fold expression changes with absolute difference (Δ) values in fragments per kb of transcript per million mapped reads (FPKM) > 5.0. We found 1865 genes upregulated and 893 genes downregulated in P7.5 Kit^−^ SGs compared with P0.5 PSGs (Additional file 2: Figure S5; Additional file 5: Table S4). Also, 135 genes were upregulated and 341 genes downregulated in P7.5 Kit^+^ SGs compared with Kit^−^ SGs (Additional file 2: Figure S5; Additional file 5: Table S4). On average, the expression changes observed between P0.5 PSGs and P7.5 Kit^−^ SGs were greater than those between Kit^−^ SGs and Kit^+^ SGs. A GO analysis of each set of differentially expressed genes using the Database for Annotation, Visualization and Integrated Discovery (DAVID) tool [54] identified terms such as “cell cycle” and “transmembrane receptor protein tyrosine kinase signaling pathway” (Additional file 6: Table S5).

Because many of the DMRs of clusters 1 and 2 are likely to be regulatory regions (see above), we next sought to correlate these DMRs with gene expression changes. Of the 1150 genes physically linked to the cluster-1 DMRs (identified by GREAT), 182 showed expression changes (>2.0-fold and ΔFPKM > 5.0) (99 up and 83 down) between P0.5 PSGs and P7.5 Kit^−^ SGs, and 64 (17 up and 47 down) between P7.5 Kit^−^ and Kit^+^ SGs (Additional file 2: Figure S5). Of the 2772 genes linked to the cluster-2 DMRs, 457 showed expression changes (245 up and 212 down) between P0.5 PSGs and P7.5 Kit^−^ SGs, and 162 (14 up and 148 down) between P7.5 Kit^−^ and Kit^+^ SGs (Additional file 2: Figure S5). Overall, the probability of showing an expression change was significantly greater for the DMR-linked genes than for the entire gene set (*P* < 1.0 × 10^−6^ by chi-squared test).

**Fig. 5:**
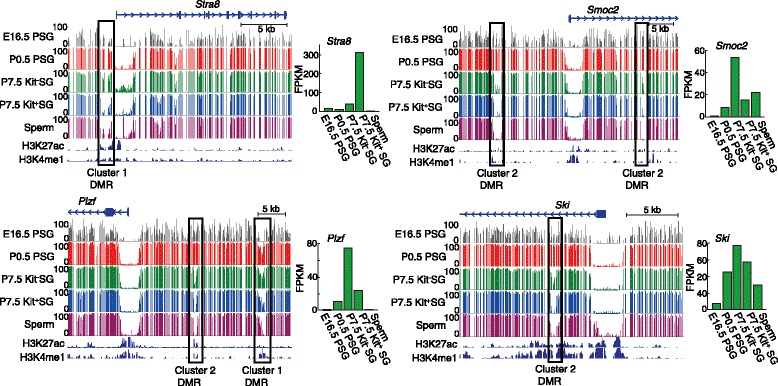
Examples of the DMRs linked with gene expression. Representative CG methylation profiles are shown for the DMRs located near or within genes essential for spermatogenesis (*Stra8* and *Plzf*) or those showing high expression in P7.5 Kit^−^ SGs (*Smoc2* and *Ski*). Histone modification data (H3K27ac and H3K4me1) from the adult mouse testis [50] are also shown. Gene expression levels are shown on the right of each methylation profile. The DMRs are marked with open squares

Examples of the DMRs with the methylation changes of the genomic region and expression changes of the linked gene are shown in Fig. 5. *Stra8* is inducible by retinoic acid and important for the transition into meiosis [55, 56]. This gene had a cluster-1 DMR in the upstream region, consistent with its highest expression in P7.5 Kit^+^ SGs, and the DMR had high levels of the enhancer histone marks such as H3K4me1 and H3K27ac in adult testis (Fig. 5), suggesting that the DMR has a regulatory function. *Plzf* encodes a transcriptional regulator required for SSC maintenance [8]. This gene had a cluster-1 and a cluster-2 DMR in the upstream region, the latter of which is consistent with its strongest induction in Kit^−^ SGs. Many other genes involved in the signal transduction pathways for SSC self-renewal (*Foxo1, Ret, T,* and *Cxcr4*) also had DMRs of clusters 1 and 2 within or around the genes (within 50-kb upstream and 50-kb downstream) and showed high levels of gene expression in Kit^−^ SGs (Additional file 2: Figures S1 and S6). Again, the enhancer marks were found in the DMRs.

There were also genes showing high levels of expression in P7.5 Kit^−^ SGs, but with no reported role so far in SSCs or during spermatogenesis (Fig. 5). *Smoc2* encodes a member of the SPARC family matricellular calcium-binding proteins and is highly expressed during embryogenesis and wound healing [57]. This gene had two cluster-2 DMRs, one in the upstream region and the other in an intron, and showed highest expression in Kit^−^ SGs, consistent with its possible role in stem cells [58]. *Ski* is a proto-oncogene encoding a repressor of transforming growth factor beta activity [59]. This gene had a cluster-2 DMR in an intron and showed expression throughout male germ cell development, with its highest expression in Kit^−^ SGs. Because *Ski* expression precedes demethylation of the DMR, its changed methylation status might be secondary. Nevertheless, these results suggest that the combined use of WGBS and RNA-seq can detect potentially important genes and regulatory regions. Interestingly, most DMRs of clusters 1 and 2 remained hypomethylated in the subsequent stages irrespective of gene activity, leaving the epigenetic signatures in the sperm genome (Figs. 4a and 5).

We previously showed that forced expression of *Dnmt3b* induces *Kit* expression and that *Uhrf1/Np95* (involved in maintenance methylation) ablation interferes with the Kit- SG to Kit + SG transition [23]. This suggests that DNA methylation is important for this transition. Thus cluster-3 DMRs, which gain methylation in Kit + SGs, may be of interest. We therefore looked for genes that are associated with cluster-3 DMRs and show a more than 2-fold expression change in this transition. As a result, three genes were identified (*Col22a1*, *Gfra1*, and *Pcp4l1*), among which *Gfra1* encoding a component of the GDNF receptor essential for SSC maintenance (Additional file 2: Figure S6) showed eleven-fold upregulation. The cluster-3 DMR of *Grfa1* was located 40-kb downstream of its 3′ end and contained some evolutionarily conserved sequences, suggesting a regulatory role. This may be consistent with the previously observed upregulation of *Gfra1* in *Dnmt3l* knockout SSCs [60] although it is not clear whether this dysregulation involves impaired methylation.

### The DMRs are Rich in binding motifs for specific transcription factors

**Fig. 6:**
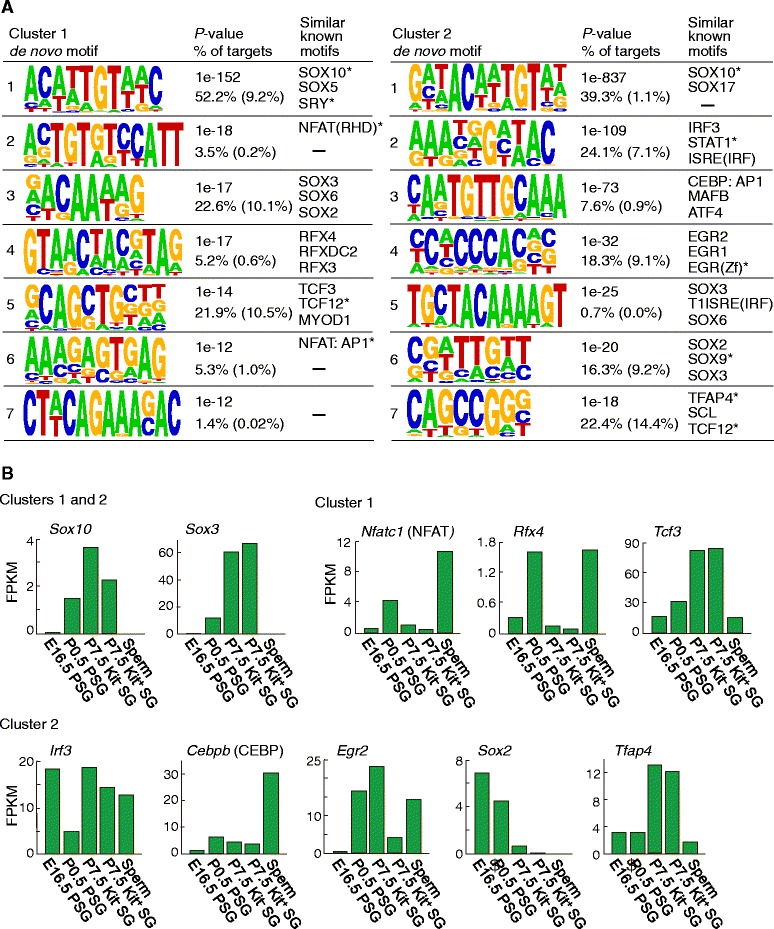
Motifs enriched in the DMRs. **a** Enriched sequence motifs identified by the HOMER de novo motif analysis of the cluster-1 and −2 DMRs. The top seven de novo motifs are shown. The fraction containing at least one instance of each motif is given under the *P* value, with the expected frequency of the motif in random background regions given in parentheses. The closely matched known motifs are shown on the right (*top three*, similarity score > 0.6). The motif symbols marked with asterisks are sourced from *Homo sapiens*. **b** Expression dynamics of messenger RNAs encoding the transcription factors bound to the known motifs in (**a**). The expression dynamics of the top-ranked transcription factors are shown

Finally, we sought to identify transcription factor binding motifs in the DMR sequences of clusters 1 and 2 by the Hypergeometric Optimization of Motif Enrichment (HOMER) analysis [61]. Short sequence motifs enriched in the DMRs of these clusters were first identified, and known transcription factor binding motifs similar to these “de novo motifs” were discovered. We found that the identified motifs were highly similar to the binding motifs for SOX family members such as SOX10 and SOX3 (Fig. 6a). The critical function of SOX3 in SG differentiation has been established [62]. In contrast, the role of SOX10 is not known for SSCs or in spermatogenesis although it does have a role in the maintenance of neural crest stem cells and the melanocyte lineage [63, 64]. The RNA-seq data showed that *Sox3* was highly expressed in P7.5 Kit^−^ and Kit^+^ SGs and that *Sox10* was also expressed at lower levels (Fig. 6b).

In addition to the SOX family members, the cluster-1 DMRs showed enrichment in motifs similar to the binding motifs for transcription factors NFATC1 (cytokine induction), RFX4 (transactivation), and TCF3 (also known as E2A) (morphogenesis) (Fig. 6a). The cluster-2 DMRs showed motif enrichment for IRF3 (interferon response), CEBPB (CEBPB/AP1 complex; immune response), EGR2 (cell proliferation, neural development), SOX2 (pluripotency), and TFAP4 (cell proliferation, differentiation blocking) (Fig. 6a). Many of these transcription factors were expressed in P0.5 PSGs or P7.5 SGs (Fig. 6b; Additional file 2: Figure S7). The cluster-1 and −2 DMRs associated with *Stra8*, *Plzf*, *Smoc2*, or *Ski* (Fig. 5) contained these motifs multiple times, as well as those similar to the binding motifs for SOX10 and SOX3 (Additional file 2: Figure S8). Although the significance of these motifs and transcription factors in SSC function and/or spermatogenesis awaits further studies, our results show that local methylation changes can identify likely candidates for regulatory regions.

## Discussion

The neonatal and early postnatal stages are important for the establishment of the SSC system in the male germline. It is well established that epigenetic mechanisms play crucial roles in mammalian germline development [16], but how they contribute to SSC derivation and differentiation has not been addressed yet. In this study, we have performed WGBS and RNA-seq on neonatal PSGs and early postnatal SGs including the nascent SSC population and compared the results with those from fetal PSGs and adult spermatozoa. Below, we discuss our major findings with reference to the recently published WGBS and RNA-seq data from adult germ cells [24].

First, we identified PMDs in these germ cells, which are similar to those found in cultured human fibroblasts [33, 34], human cancer cells [35, 36], and human placenta [37]. The germline PMDs share features with those identified in other cell types: localization in genomic regions with low GC contents, low CGI density, low gene density, and overlap with cLADs. Furthermore, the genes in the PMDs were silenced, just as were those found in cancer cells [35, 36], sharing functional features. Since the PMDs in cancer cells are marked by repressive histone modifications such as H3K27me3 and H3K9me3 [36], genes located in the regions may be silenced through these modifications. We speculate that a similar silencing mechanism may operate in the germline PMDs because they share genomic localizations and structural features with the cancer PMDs. The PMDs were not seen in E10.5 primordial germ cells, but arose during the early phase of global de novo methylation in fetal PSGs, and persisted to the adult sperm stage. We confirmed the existence of PMDs in adult male germ cells, including the SSC-rich population (Thy1^+^), using published data [21]. The mechanisms of PMD formation and the biological significance of PMDs are yet to be determined, but considering their association with cLADs, we speculate that germ cells, cancer cells, and placenta may have something in common in their nuclear architecture and/or function.

Second, we found high levels of CH methylation and 5hmCs in neonatal PSGs. The CH methylation occurred globally, but predominantly in non-PMD regions, during the wave of de novo methylation in fetal PSGs arrested at G1/G0. CH methylation reached the highest level in neonatal PSGs and disappeared after the resumption of mitosis. This confirmed and extended the previously observed CH methylation dynamics at SINE B1 copies [42]. Although we do not know the biological significance of this transient CH methylation, its accumulation is likely associated with the high de novo methylation activity and arrested cell cycle state of PSGs, as discussed previously [27]. So far, CH methylation has been reported in embryonic stem cells, brain tissues, and oocytes [27, 33, 65], but neonatal PSGs showed the highest level. In fact, two-thirds of all 5mCs of this cell type occurred at CH sites. The extremely high level of CH methylation and subsequent loss in early SGs help explain previous immunocytological observations, including the replication-dependent loss of 5mC signals [22]. In contrast to the CH methylation dynamics, CG methylation showed less changes (except for the stage-specific DMRs) during the neonatal and early postnatal stages. In particular, we confirmed the stable maintenance of CG methylation at the paternally methylated ICRs and retrotransposons. Thus, our study resolved the question previously posed by immunocytology regarding the maintenance of epigenetic imprints. As for 5hmC, we confirmed previous immunocytological observations that chromosome satellite repeats are enriched in 5hmC in neonatal PSGs [45, 46] (Additional file 2: Figure S4). Interestingly, certain retrotransposons, such as IAP, L1MdA, and L1MdT, which are resistant to CG methylation reprogramming (demethylation) in primordial germ cells, had high levels of 5hmC and low levels of CH methylation in neonatal PSGs. This suggests that reprogramming-resistant regions accumulate 5hmC in primordial germ cells but do not undergo complete demethylation beyond this intermediate stage. Also, the de novo DNMTs responsible for CH methylation introduce few CH methylations in such reprogramming-resistant regions that maintain residual CG methylation.

Third, we identified > 5000 stage-specific DMRs showing developmental stage-specific changes (>30 %) in CG methylation between P0.5 PSGs and P7.5 SGs or between P7.5 Kit^−^ and Kit^+^ SGs, despite the small differences (<0.6 %) in global CG methylation. We focused on the ~2800 DMRs that showed reduced methylation in P7.5 Kit^−^ or Kit^+^ SGs (cluster-1 and −2 DMRs) and found that many of them are located in intergenic regions or introns (not promoters), have histone marks specific for enhancers, and show association with specific gene functions such as cell proliferation, cell movement, stem cell function, and spermatogenesis. Such limited usage of differential promoter methylation for gene regulation has been observed in other developmental contexts, such as the lineage specification of human embryonic stem cells [66], highlighting that this phenomenon is not specific to male germ cell development. Approximately 700 DMR-linked genes showed expression changes (>2.0-fold) between the stages, implying that the DMRs are indeed regulatory regions. The fact that most developmentally regulated DMRs map to distant cis regulatory regions rather than promoters fits well with the general landscape where DNA methylation is preferentially established in intergenic regions in male germ cells, while gene bodies and some CG-rich promoters are the favorite targets of methylation in female germ cells [28, 67]. Interestingly, most DMRs of clusters 1 and 2 stayed hypomethylated in the subsequent stages of spermatogenesis irrespective of gene activity, leaving the epigenetic signatures (hypomethylation) in the sperm genome. In early postnatal stages, de novo methylation occurred mainly in the PMDs and the DMRs of clusters 5 and 6, most of which were mapped in the PMDs. In this period, Dnmt3l was strongly downregulated (Additional file 2: Figure S1), suggesting that the de novo methylation is independent of DNMT3L. It is also possible that there may be a switch from DNMT3A to DNMT3B during this period, considering the changes in mRNA levels. However, targeted disruption of Dnmt3b in the male germline does not cause any phenotype [21], which seems consistent with the fact that the genes in the PMDs were consistently silent. A previous study on undifferentiated and differentiating adult SGs showed that methylation differences at promoters are rare (only seven promoters showed changes) [24]. We found only 10 promoter DMRs using the same criteria, confirming these observations in adult SGs. However, none of the seven promoters identified in the previous adult SSC study [24] coincided with our DMRs, perhaps because of the differences in developmental stage (adult versus early postnatal stage), cell markers used (Thy1^+^ and Kit^−^ for the SSC-enriched population), or both.

Finally, the identified DMRs were extremely rich in binding motifs for specific transcription factors such as the SOX family members. The SOX family transcription factors are well-established regulators of cell fate decisions during development, with additional roles in adult tissue homeostasis and regeneration [68]. The role of SOX3 in SG differentiation has been established [62], but other SOX members expressed in early postnatal SGs might also play roles in SSC formation and differentiation. In addition to the SOX binding motifs, we identified potential binding motifs for many other transcription factors, some of which have a role in development or cell proliferation. It will be interesting to explore the role of these and related factors in SSC formation and/or differentiation.

## Conclusions

Our WGBS and RNA-seq analyses revealed the existence of PMDs, a unique accumulation and distribution of CH methylation and 5hmC marks in neonatal PSGs, and stage-specific DMRs rich in specific transcription factor binding motifs. These findings contrast with the reported scarcity of differential methylation in adult SSCs differentiation and represent a unique phase of male germ cell development. Although the roles of the identified motifs and transcription factors in SSC function and/or spermatogenesis await further studies, our results show that local methylation changes can identify potential regulatory regions in developing germ cells. The data presented here will serve as an important resource for future studies, and our results provide important insights into the epigenetic regulation of SSC formation and differentiation.

## Methods

### Mouse husbandry

C57Bl/6 mice and *Oct4*-EGFP transgenic mice [11] maintained on a C57Bl/6 background were housed and all experiments were performed under the ethical guidelines of Kyushu University, Yokohama City University, and Tokyo University of Agriculture.

### Preparation of germ cells

PSGs and SGs were respectively isolated from P0.5 and P7.5 testes of *Oct4*-EGFP mice [11] by fluorescence-activated cell sorting as described [69]. Briefly, testes were incubated in phosphate-buffered saline supplemented with 1 mg/ml collagenase (Sigma-Aldrich) and 100 units of DNase I (Invitrogen) for 15 min at 37 °C with agitation. For Kit staining, suspended cells were incubated at 4 °C with Fc-block for 15 min and then with an APC-anti-Kit antibody (eBiosciences) for 30 min. Propidium iodide (2 μg/ml; Sigma-Aldrich) was added, and cells were sorted using MoFlo (Beckman-Coulter). The purity of the sample was checked by the expression of some marker genes (e.g., *Oct4*, *Kit,* and *Plzf*) using quantitative polymerase chain reaction. Isolation of E16.5 PSGs and adult spermatozoa was performed as previously described [18, 28].

### WGBS and analysis

WGBS was done with two biological replicates for all cell types. DNA was extracted from 4–9 × 10^4^ cells using the AllPrep DNA/RNA Mini Kit (Qiagen) according to the manufacturer’s instructions. Libraries were prepared from 100 ng of sample DNA spiked with 1 ng of unmethylated lambda phage DNA (Promega) using the PBAT method [26, 27]. Concentrations of the PBAT products were quantified using the KAPA Illumina Library Quantification Kit (Kapa Biosystems). The libraries were sequenced on an Illumina HiSeq 2000 (HiSeq Control Software (HCS) version 1.5.15 and Real Time Analysis (RTA) version 1.13.48) or an Illumina HiSeq 2500 (HCS version 2.0.5 and RTA version 1.17.20) to generate 118-nucleotide (nt) single-end reads.

We obtained high-quality reads after trimming the low-quality bases from the 3′ end and the adapter sequences from the 5′ end using the NGS QC toolkit [70]. The resulting reads were aligned to the reference mouse genome (mm10) using Bismark alignment software (version 0.10.0) [71]. We used parameters of 28 for the seed length, 1 for the maximum number of mismatches permitted in the seed, and the option “--pbat” that works for PBAT libraries. Only uniquely aligned reads were analyzed. We estimated the bisulfite conversion rate using reads that were aligned uniquely to the lambda phage genome. Counts from the symmetric cytosines were combined for strand-independent analysis of CG methylation. We subsequently analyzed CG sites covered at least six times, CH sites covered at least four times, and discarded cytosines covered by more than 100 reads.

RefSeq genes and repeat element annotations (RepeatMasker) were downloaded from the UCSC genome browser database (mm10) [72]. Repeat consensus sequences were downloaded from Repbase [73]. The PMDs were identified using MethylSeekR [38].

To identify stage-specific DMRs, the methylation level of each 500 bp window was determined from ≥ 5 CG sites covered ≥ 10 times. The window was identified as a DMR if the difference exceeded 30 % and was shown significant by two-sample *t*-tests with unequal variance after correction for multiple hypotheses testing using the Benjamini–Hochberg method. Overlapping DMR windows (sliding steps of 100 bp) were merged. A cluster analysis of the identified DMRs was performed using R software with the complete linkage method (default). The CG methylation levels in the DMRs in P0.5 PSGs and the changes up to the P7.5 Kit^+^ SG stage were used as the input dataset.

### Oxidative bisulfite sequencing and analysis

Libraries were prepared from bisulfite-converted DNA and oxidized plus bisulfite-converted DNA, each from two biological replicates. Each library was prepared from 30 ng P0.5 PSG DNA with 0.3 ng spike-in conversion controls. The DNA was sonicated to 500 bp using the S220 Focused-ultrasonicator (Covaris) before spiking in. Oxidation reactions and bisulfite conversions were performed using TrueMethyl kits (Cambridge Epigenetix), and then oxidized and bisulfite-converted DNA or simply bisulfite-converted DNA was added with adapters using the PBAT method as described previously [26, 27]. The spike-in control was prepared using the protocol of 5hmC TAB-Seq kits (Wisegene). Trimmed high-quality reads were aligned to the reference mouse genome (mm10) or to the consensus major and minor satellite repeat sequences. The level of 5hmC was calculated by subtracting the value obtained from the oxidation plus bisulfite treatment dataset from that obtained from the corresponding bisulfite-only dataset.

### RNA-seq and analysis

Two replicate preparations were analyzed for all cell types. Total RNA was extracted from 4–9 × 10^4^ cells using the AllPrep DNA/RNA Mini Kit (Qiagen) or RNeasy Micro Kit (Qiagen) according to the manufacturer’s instructions. Thirty to 100 ng total RNA was subjected to enrichment for poly(A)^+^ RNA and libraries were prepared using the TruSeq RNA Sample Prep Kit v2 (Illumina). For E16.5 PSGs, 10 ng of total RNA was used to synthesize and amplify double-stranded cDNAs using the SMARTer Ultra Low RNA kit (Clontech). The libraries were amplified for 15 cycles and then sequenced on an Illumina GAIIx instrument to generate 36-nt single-end reads or on an Illumina HiSeq 2000 instrument to generate 100-nt paired-end reads.

RNA-seq reads were mapped to the mouse mm10 genome assembly using TopHat (version 2.0.8) [74]. The mapped reads from two biological replicates were subsequently analyzed by Cufflinks (version 2.1.1) to estimate the transcript abundance [75], and the expression level of each transcript was indicated as a FPKM value.

### GO analysis

The GO enrichment for stage-specific DMRs was done using GREAT (version 2.0) [53]. The FDR cutoff was 0.05 and the fold enrichment was kept at 2. Genes linked to the DMRs were selected using basal plus extension criteria with a default setting (proximal, from 5 kb upstream to 1 kb downstream of the transcription start site; distal, up to 1 Mb from the transcription start site), assuming that the DMRs were either proximal or distal regulatory regions. The GO analysis for differentially expressed genes was done using DAVID [54] with an all-mouse gene background and the FDR cutoff at 0.1.

### Motif analysis

We searched for de novo motifs enriched in each DMR cluster using the HOMER tool [61]. We used default parameters with a fragment size of 200 bp and with “-mask” parameter to use the repeat-masked sequences. Random background regions that matched the GC-content distribution of the input DMR sequences were used as controls. The motif search for each DMR sequence was performed using the position weight matrix file of de novo motifs or known motifs from the motif database included in the HOMER tool with a relative profile score threshold > 90 %.

### Immunostaining

For 5mC and 5hmC immunofluorescence staining, chromosomes fixed on slides were incubated in 4 N HCl at 37 °C for 30 min. After neutralization in 100 mM Tris–HCl (pH 7.5) for 10 min, the specimens were incubated with anti-5mC (mouse monoclonal; Calbiochem) and/or anti-5hmC (rabbit polyclonal; Active Motif) antibodies. After blocking with 1 % bovine serum albumin and 0.2 % Triton X-100 in phosphate-buffered saline, the specimens were incubated with anti-mouse and/or anti-rabbit secondary antibodies coupled with Alexa Flour 488 or 594 (Life Technologies) at room temperature for 30 min. The samples were then counterstained with DAPI in the mounting medium.

### Data from other sources

Previously published WGBS data from mouse E13.5 primordial germ cells and E16.5 PSGs [18] and RNA-seq data from mouse adult spermatozoa [28] were downloaded and remapped to allow comparison with the current data. CGI data were taken from Illingworth et al. [76]. Previously published H3K27ac and H3K4me1 ChIP-seq data from the adult mouse testis [50] and cLAD data [77] were downloaded from the NCBI Gene Expression Omnibus database.

### Data access

Sequence data have been deposited in DDBJ/GenBank/EMBL under accession numbers DRA002477 and DRA002402.

## Additional files

Additional file 1: Table S1. Sequencing runs and mapping summary. (XLSX 47 kb) Additional file 2: Figures S1–S8.
**Figure S1:** Expression of key molecular markers in isolated cells. **Figure S2:** CG methylation at ICRs and retrotransposons. **Figure S3:** PMD distribution in each chromosome. **Figure S4:** Replication-dependent loss of 5mC and 5hmC in neonatal spermatogonia after the resumption of mitosis. **Figure S5:** Changes in gene expression levels between stages. **Figure S6:** Genes involved in the signal transduction pathways for SSC self-renewal have stage-specific DMRs. **Figure S7:** Expression dynamics of transcription factors listed by motif analysis of the DMRs. **Figure S8:** Motifs found in the representative DMRs. (PDF 26886 kb) Additional file 3: Table S2. Locations and methylation levels of the DMRs. (XLSX 437 kb) Additional file 4: Table S3. GO terms and genes obtained by the GREAT analysis. (XLSX 33 kb) Additional file 5: Table S4. Expression levels of the differentially expressed genes. (XLSX 278 kb) Additional file 6: Table S5. GO terms enriched for the differentially expressed genes identified by DAVID analysis. (XLSX 78 kb)

## Abbreviations

5mC: 5-methylcytosine
5hmC: 5-hydroxymethylcytosine
CGI: CG island
DMR: Differentially methylated region
EGFP: Enhanced green fluorescent protein
FPKM: Fragments per kilobase of exon per million mapped fragments
GO: Gene ontology
IAP: Intracisternal A particle
ICR: Imprinting control region
LINE: Long interspersed nuclear element
LTR: Long terminal repeat
PBAT: Post-bisulfite adaptor tagging
PGC: Primordial germ cell
PMD: Partially methylated domain
Pol2: RNA polymerase II
PSG: Prospermatogonia
SINE: Short interspersed element
SG: Spermatogonia
SSC: Spermatogonial stem cell
WGBS: Whole-genome bisulfite sequencing
