# Supplementary material for: DNA methylation and gene expression dynamics during spermatogonial stem cell differentiation in the early postnatal mouse testis
Source: BMC Genomics. 2015 Aug 20;16(1):624. doi: 10.1186/s12864-015-1833-5 (PMC4546090; doi:10.1186/s12864-015-1833-5)
Supplement: Additional file 2: Figures S1–S8. — Figure S1: Expression of key molecular markers in isolated cells. Figure S2: CG methylation at ICRs and retrotransposons. Figure S3: PMD distribution in each chromosome. Figure S4: Replication-dependent loss of 5mC and 5hmC in neonatal spermatogonia after the resumption of mitosis. Figure S5: Changes in gene expression levels between stages. Figure S6: Genes involved in the signal transduction pathways for SSC self-renewal have stage-specific DMRs. Figure S7: Expression dynamics of transcription factors listed by motif analysis of the DMRs. Figure S8: Motifs found in the representative DMRs. (PDF 26886 kb) [file 12864_2015_1833_MOESM2_ESM.pdf]

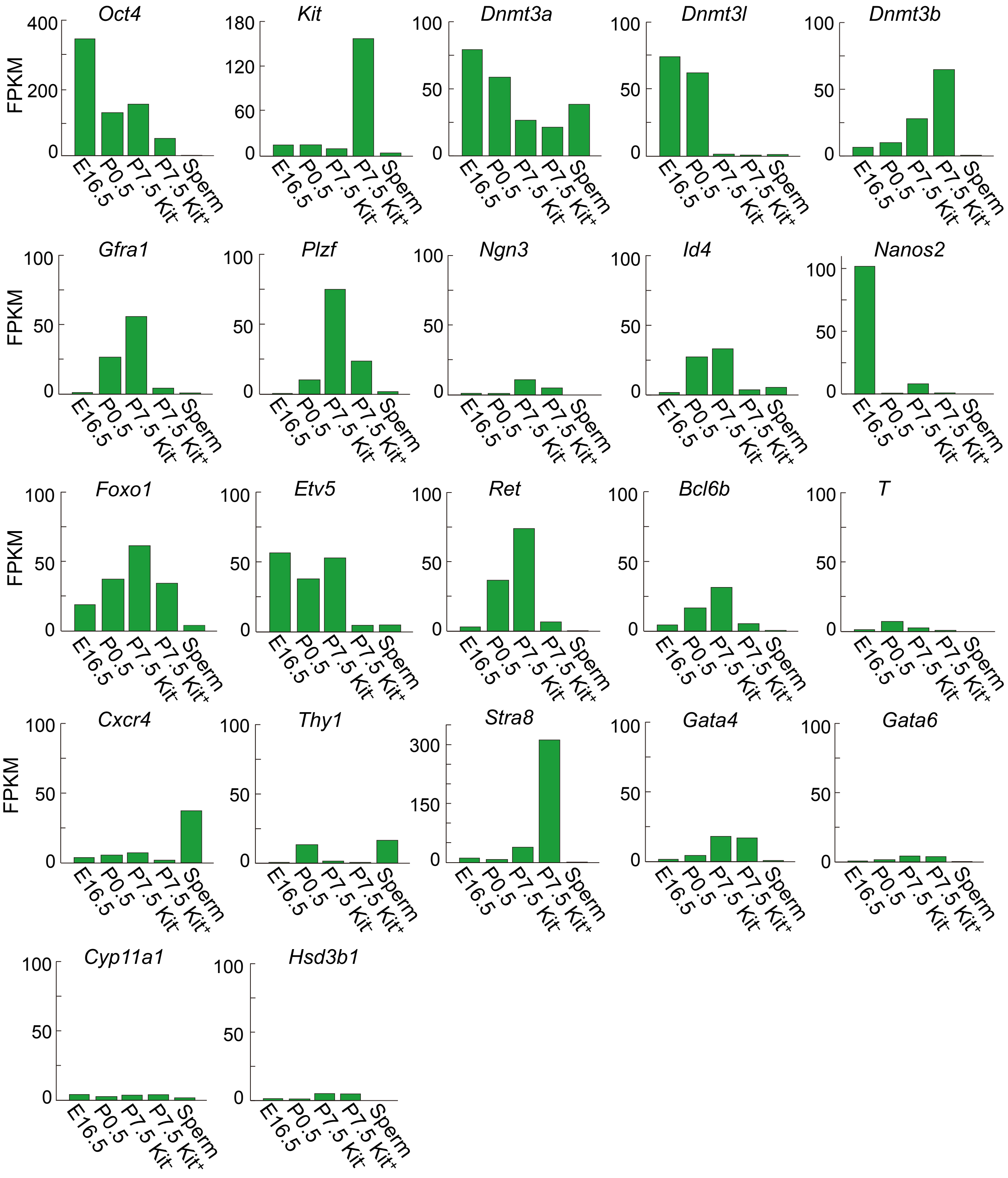

Figure S1. Expression of key molecular markers in isolated cells. Expression dynamics of the genes encoding known markers, epigenetic regulators, and signal transduction pathway components in isolated cells. Expression of the genes encoding Sertoli cell (*Gata4* and *Gata6*) and Leydig cell markers (*Cyp11a1* and *Hsd3b1*) is also shown. Adult sperm RNA-seq data are from Kobayashi et al. (2012).

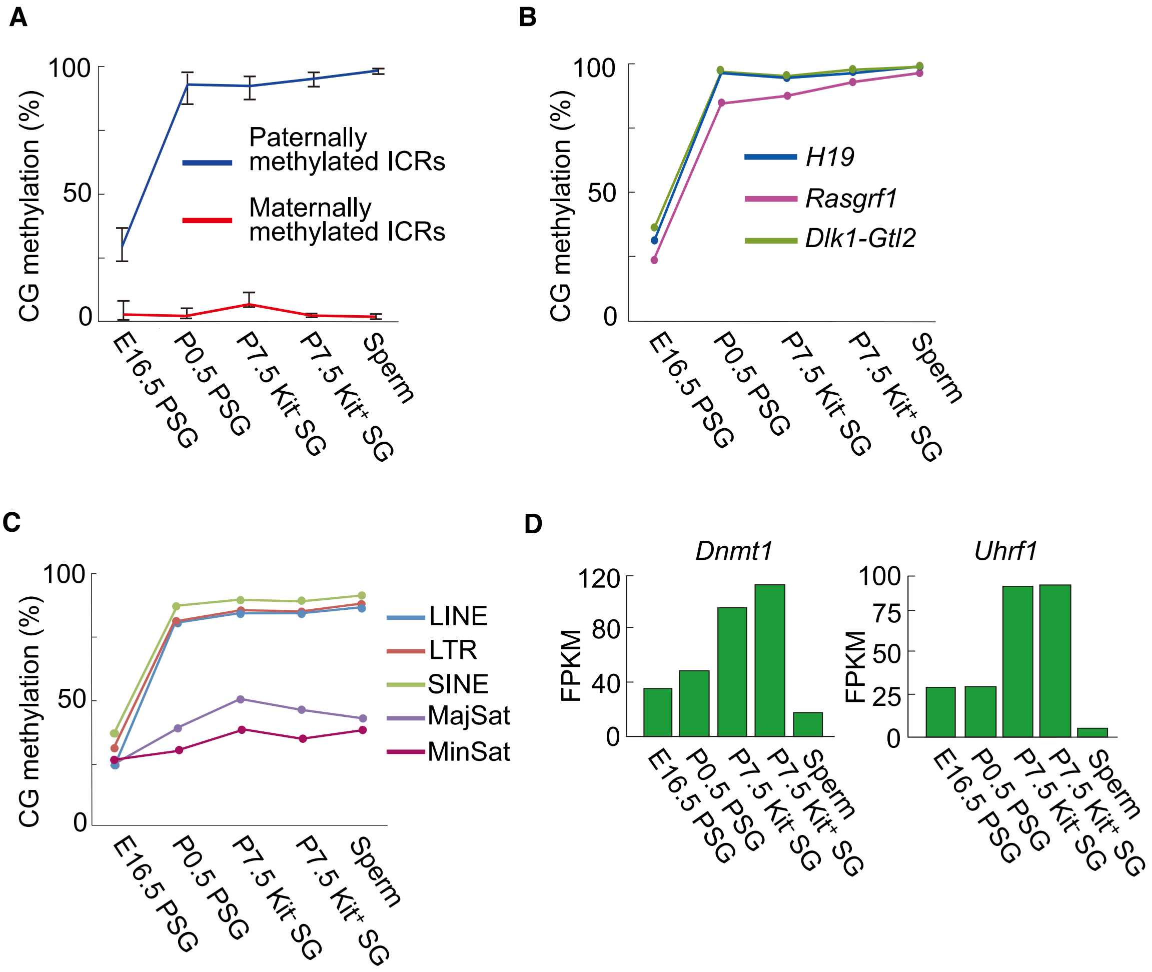

Figure S2. CG methylation at ICRs and retrotransposons. (A) The mean levels of CG methylation at the paternally and maternally methylated ICRs in the five developmental stages studied. Three paternally methylated ICRs (*Dlk1-Gtl2*, *H19*, and *Rasgrf1*) and 12 maternally methylated ICRs (*Nespas-Gnasxl*, *Gnas* exon 1A, *Peg10*, *Mest*, *Peg3*, *Snrpn*, *Kcnq1ot1*, *Zac1*, *Grb10*, *U2af1-rs1*, *Igf2r,* and *Impact*) (Tomizawa et al. 2011) were analyzed. The mean (line graph), maximum, and minimum methylation levels (whiskers) are shown. **(B)** Levels of CG methylation at each of the three paternally methylated ICRs. **(C)** Levels of CG methylation at repeat sequences. The levels at the major and minor satellite repeats were calculated from the reads aligned to their consensus sequences, and those at the retrotransposons were calculated from the reads aligned to the reference mouse genome (mm10). **(D)** Expression dynamics of genes that have important roles in CG methylation maintenance.

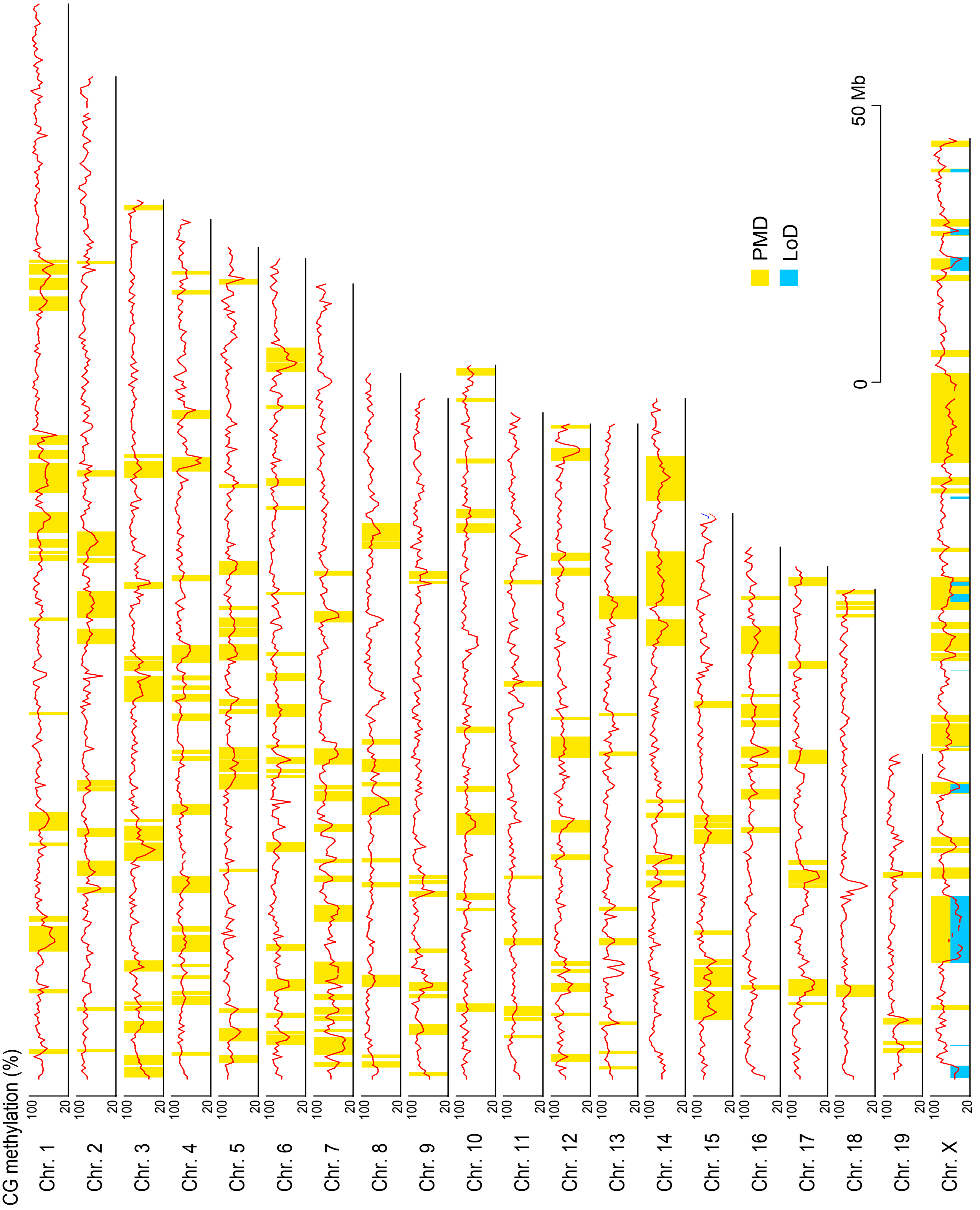

Figure S3. **PMD distribution in each chromosome.** The large PMDs (≥ 500 kb) determined in P0.5 PSGs are highlighted in yellow. The CG methylation levels in P0.5 PSGs are also shown (red lines). Regions highlighted in blue on the X chromosome indicate the LoDs reported previously (Ikeda et al. 2013).

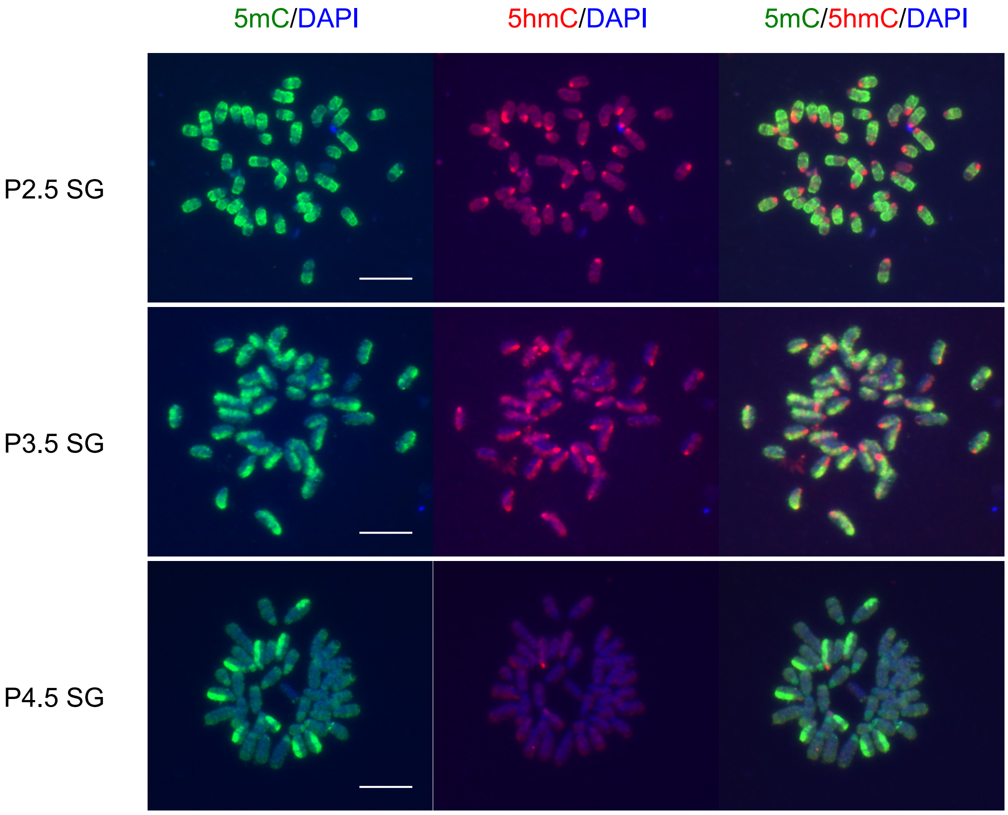

Figure S4. **Replication-dependent loss of 5mC and 5hmC in neonatal spermatogonia after the resumption of mitosis.** Chromosomes were prepared from P2.5-P4.5 SGs (presumably Kit^{-}), and immunofluorescence staining was performed with 5mC (green) and 5hmC antibodies (red). DNA was stained with DAPI (blue). Bar=10 μm.

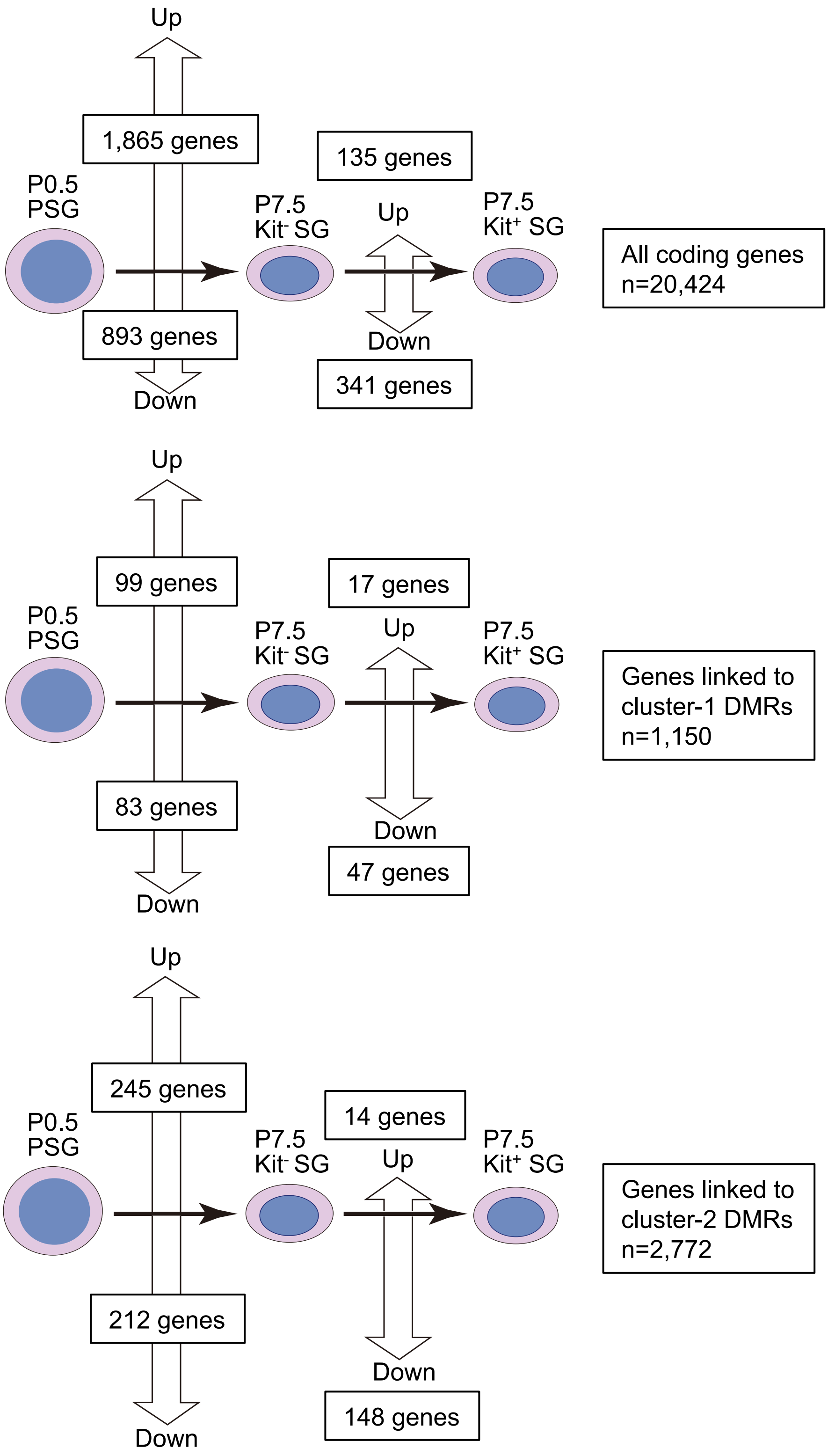

Figure S5. Changes in gene expression levels between stages. Changes in gene expression levels at the transitions from P0.5 PSGs to P7.5 Kit$^-$ SGs and from P7.5 Kit$^-$ SGs to Kit$^+$ SGs are shown schematically with the number of coding genes showing each change. Genes showing > 2.0-fold change with ΔFPKM > 5.0 were identified as either upregulated or downregulated. The data are shown for all coding genes (top) and genes linked to the cluster-1 DMRs (middle) and the cluster-2 DMRs (bottom).

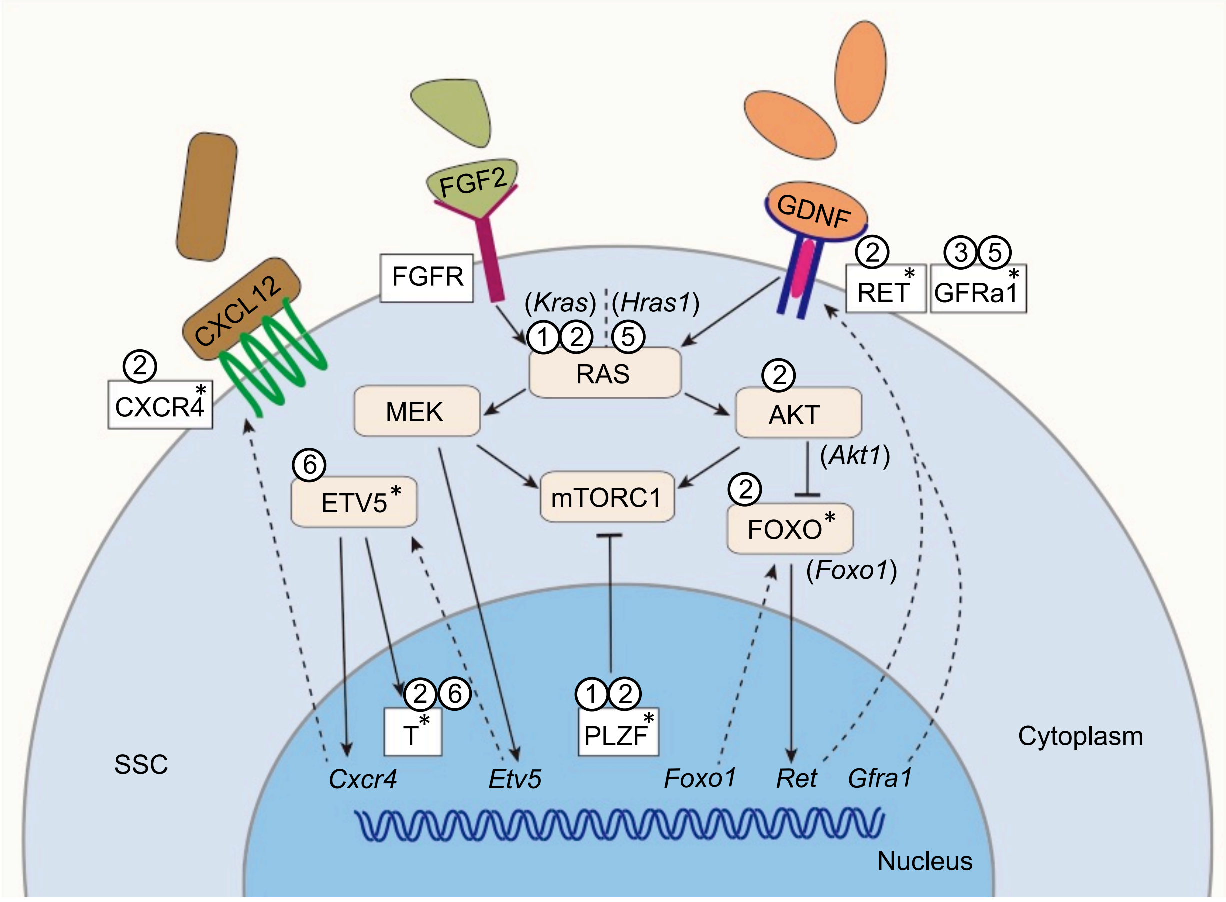

Figure S6. Genes involved in the signal transduction pathways for SSC self-renewal have stage-specific DMRs. The asterisks indicate factors that showed high expression in P7.5 Kit$^-$ SGs and downregulation in Kit$^+$ SGs in our RNA-seq data. The numbers in circles indicate the categories of DMRs (clusters 1–6) identified within 50-kb upstream and 50-kb downstream of the genes.

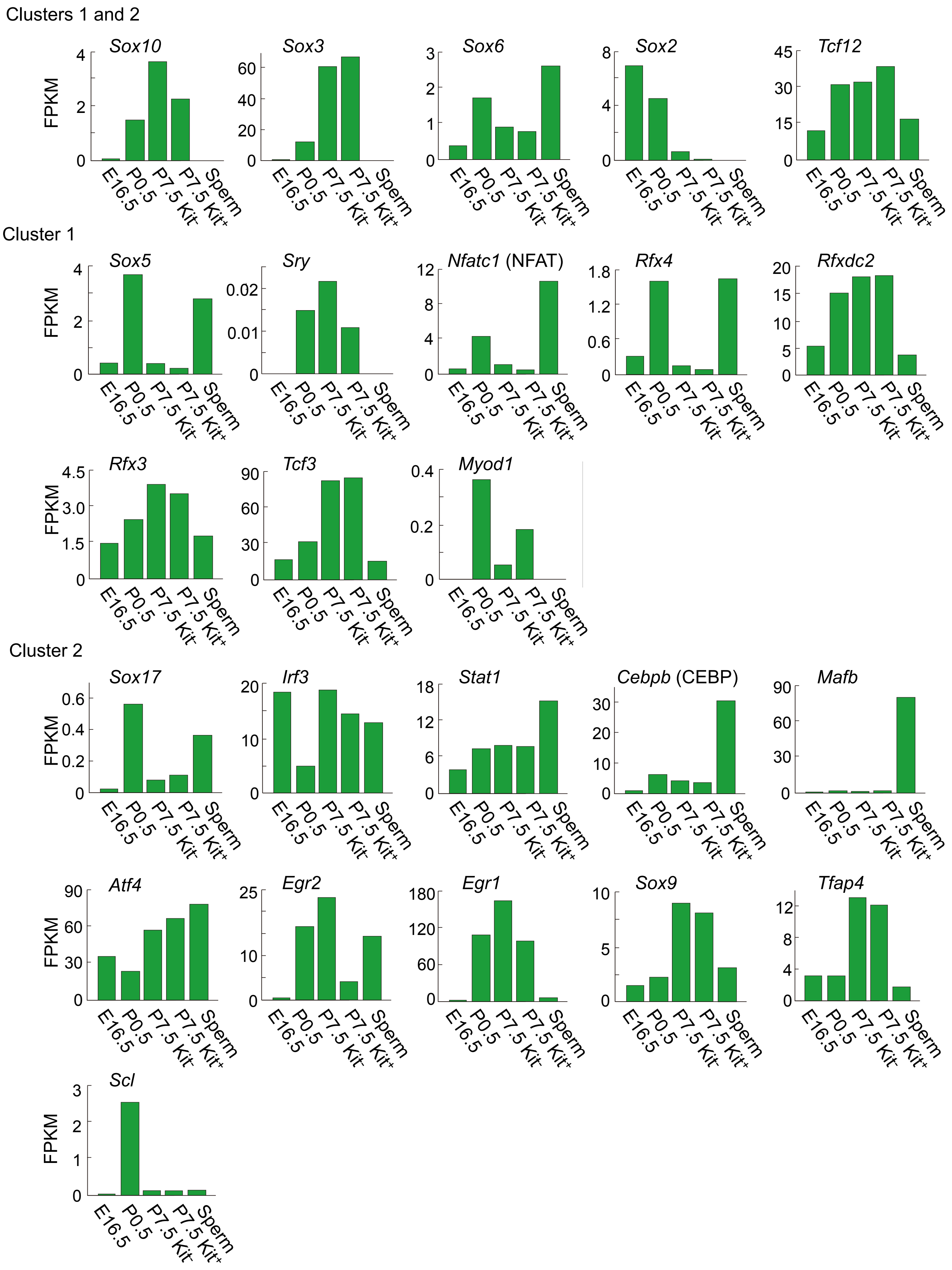

Figure S7. Expression dynamics of transcription factors listed by motif analysis of the DMRs. The expression dynamics are shown for the top three transcription factors listed by the HOMER analysis of the cluster-1 and -2 DMRs.

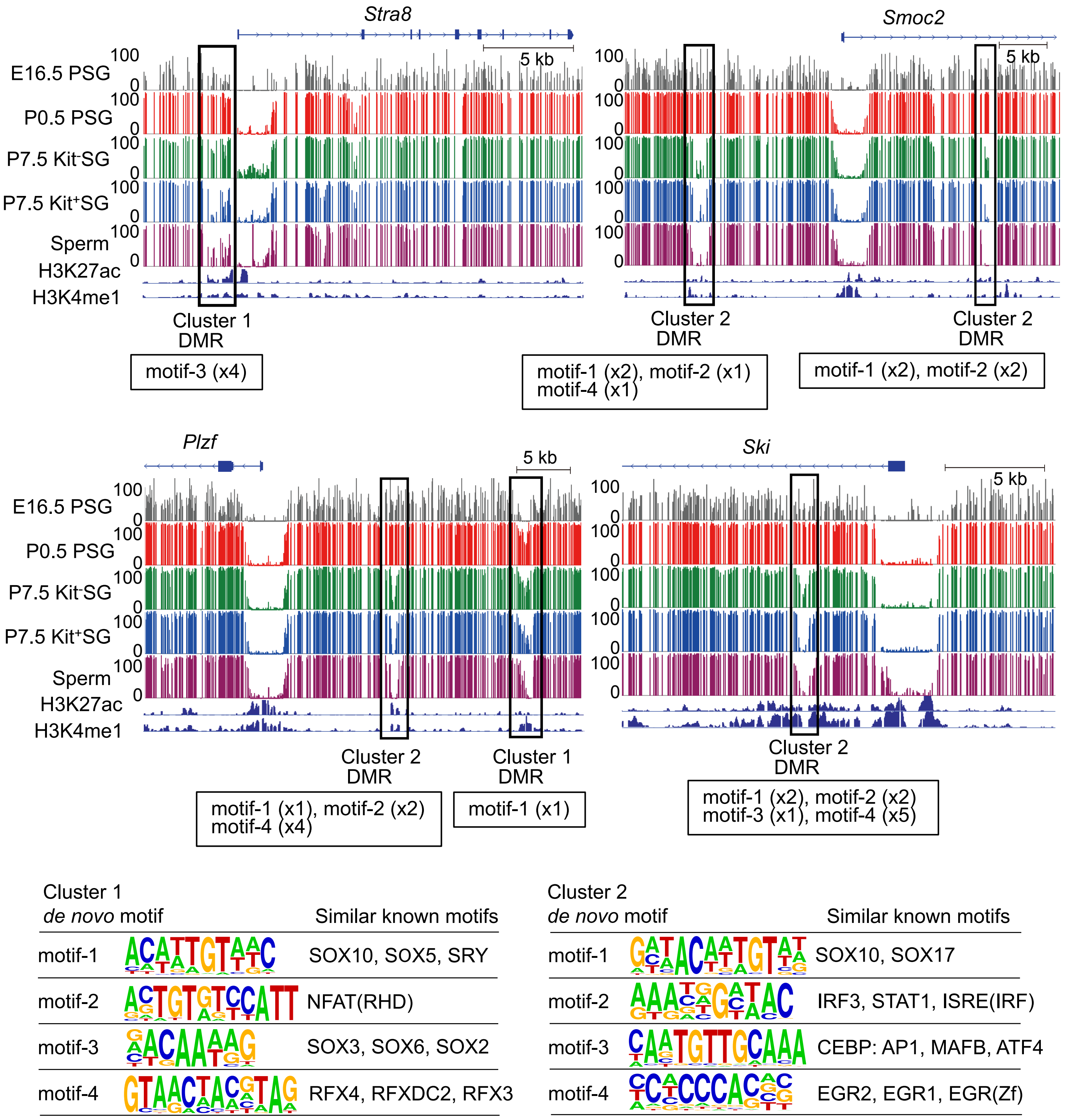

Figure S8. Motifs found in the representative DMRs. The top four de novo motifs (relative profile score > 90%; bottom, identical to the top four in Figure 6A) were assigned to each of the DMRs shown in Figure 5. The number of times that they appeared in each DMR is shown in parenthesis.
